# Rescue of Dopamine Neurons from Iron-Dependent Ferroptosis by Doxycycline and Demeclocycline and Their Non-Antibiotic Derivatives

**DOI:** 10.3390/antiox12030575

**Published:** 2023-02-24

**Authors:** Aurore Tourville, Sarah Viguier, Florencia González-Lizárraga, Rodrigo Hernán Tomas-Grau, Paola Ramirez, Jean-Michel Brunel, Mauricio Dos Santos Pereira, Elaine Del-Bel, Rosana Chehin, Laurent Ferrié, Rita Raisman-Vozari, Bruno Figadère, Patrick Pierre Michel

**Affiliations:** 1 Paris Brain Institute—ICM, Inserm, Sorbonne Université, CNRS, Hôpital Pitié Salpêtrière, 75013 Paris, France; 2 IMMCA, CONICET-UNT-SIPROSA, Tucumán 4000, Argentina; 3 UMR_MD1 Membranes et Cibles Thérapeutiques, U1261 INSERM, Aix-Marseille Université, 13385 Marseille, France; 4 Department of Basic and Oral Biology, USP, Ribeirão Preto 14040-904, Brazil; 5 BioCIS, CNRS, Université Paris-Saclay, 92290 Châtenay-Malabry, France

**Keywords:** dopamine neurons, ferroptosis, mitochondria, neuroprotection, oxidative stress, Parkinson’s disease, tetracyclines

## Abstract

Several studies have reported that the tetracycline (TC) class antibiotic doxycycline (**DOX**) is effective against Parkinson’s disease (PD) pathomechanisms. The aim of the present work was three-fold: (i) Establish a model system to better characterize neuroprotection by **DOX**; (ii) Compare the rescue effect of **DOX** to that of other TC antibiotics; (iii) Discover novel neuroprotective TCs having reduced antibiotic activity. For that, we used cultures of mouse midbrain dopamine (DA) neurons and experimental conditions that model iron-mediated oxidative damage, a key mechanism in PD pathobiology. We found that **DOX** and the other TC antibiotic, demeclocycline (**DMC**), provided sustained protection to DA neurons enduring iron-mediated insults, whereas chlortetracycline and non-TC class antibiotics did not. Most interestingly, non-antibiotic derivatives of **DOX** and **DMC**, i.e., **DDOX** and **DDMC**, respectively, were also robustly protective for DA neurons. Interestingly, **DOX**, **DDOX**, **DMC,** and **DDMC** remained protective for DA neurons until advanced stages of neurodegeneration, and the rescue effects of TCs were observable regardless of the degree of maturity of midbrain cultures. Live imaging studies with the fluorogenic probes DHR-123 and TMRM revealed that protective TCs operated by preventing intracellular oxidative stress and mitochondrial membrane depolarization, i.e., cellular perturbations occurring in this model system as the ultimate consequence of ferroptosis-mediated lipid peroxidation. If oxidative/mitochondrial insults were generated acutely, **DOX**, **DDOX, DMC,** and **DDMC** were no longer neuroprotective, suggesting that these compounds are mostly effective when neuronal damage is chronic and of low-intensity. Overall, our data suggest that TC derivatives, particularly those lacking antibiotic activity, might be of potential therapeutic utility to combat low-level oxidative insults that develop chronically in the course of PD neurodegeneration.

## 1. Introduction

Parkinson’s disease (PD) is the second-most common neurodegenerative disorder of aging. It is primarily characterized by a gradual loss of voluntary motor control resulting in typical clinical symptoms caused by dopaminergic deafferentation of the striatum, due to the death of dopamine (DA) neurons in the substantia nigra (SN) [1,2]. Studies in humans and animal models of sporadic and inherited forms of PD suggest that the progressive deterioration of vulnerable SN DA neurons may primarily arise from cellular disturbances produced by misfolding and aggregation of the synaptic protein α-Synuclein (αS), mitochondrial dysfunction or oxidative stress-mediated insults [1,3,4,5]. Such ongoing neurodegenerative events, which may mutually cooperate to promote DA cell death, appear to be amplified by brain neuroinflammatory processes in a vicious cycle of events [6,7].

The fact that several mechanisms might operate in concert to kill dopaminergic neurons suggests that compounds with multimodal neuroprotective activities might be required to tackle PD neurodegeneration [8]. In that context, we became interested in doxycycline (**DOX**), a very old antibiotic from the tetracycline (TC) family, primarily used for skin problems, including rosacea and acne vulgaris [9]. In particular, **DOX** was found to protect vulnerable DA neurons from degeneration by restraining glial cell-mediated responses in a 6-OHDA PD mouse model [10]. In addition, **DOX** was reported to prevent amyloid aggregation of αS [11] and restrain neurodegenerative changes induced by aggregated forms of αS [12]. In line with these observations, **DOX** abolished cognitive and daily life activity deficiencies in human αS A53T transgenic mice [13]. Finally, **DOX** was also reported to limit microglial inflammation induced by αS aggregated species [14].

The aim of the present study was three-fold: (i) To implement a culture model of DA neurons to explore further the nature of **DOX** neuroprotective effects; (ii) To take advantage of this culture system to compare the effects of **DOX** with those of two of its structural analogs demeclocycline (**DMC**) and chlortetracycline (**CT**) used as antibiotic medications; (iii) To demonstrate that TC derivatives designed to have minimal or no antimicrobial activity are also capable of protecting DA neurons.

Thus, we established a model system of mouse midbrain cultures in which DA neurons die spontaneously by ferroptosis, a form of regulated cell death driven by iron [15,16]. This paradigm appears particularly pertinent, as iron deposition is a typical feature of PD [8,16,17,18]. We found that **DOX** robustly protects DA neurons by limiting the production of intracellular reactive oxygen species (ROS) and preserving the function of mitochondria. **DMC** was also strongly protective but surprisingly **CT** was not. Noticeably, novel synthetic derivatives of **DOX** and **DMC**, i.e., **DDOX** and **DDMC**, respectively, which have no or minimal antimicrobial activity, respectively, remained strongly protective for DA neurons through a mechanism that seems shared with **DOX**.

## 2. Materials and Methods

### 2.1. Use of Animals

Mice used were housed, handled, and cared for in strict accordance with the European Union Council Directives (2010/63/EU). The Committee on the Ethics of Animal Experiments Charles Darwin no. 5 approved experimental protocols under authorization number Ce5/2017/005.

### 2.2. Midbrain Cell Culture Protocol

We established primary cultures of DA neurons derived from midbrain tissue of E13.5 Swiss mouse embryos (Janvier LABS; Le Genest St Isle, France) using a protocol described elsewhere [19] with some modifications. In particular, prior to mechanical trituration, midbrain tissue pieces were incubated for 20 min at 37 °C in an EDTA (2 mM)-trypsin (0.05%) solution to improve tissue dissociation. The trypsin was then neutralized with Dulbecco’s Modified Eagle’s Medium (Thermo Fisher Scientific; Courtaboeuf, France) containing 10% fetal calf serum (FCS; Biowest LLC, Les Ulis, France) and tissue trituration was performed as described before using Leibovitz L15 culture medium (Sigma Aldrich; L’lsle-d’Abeau Chesnes, France). Dissociated cells in suspension were seeded at a density of 40–60 × 10^3^ cells/cm^2^ onto Nunc 48-well multiwell plates (Roskilde, Denmark) pre-coated with 1 mg/mL polyethylenimine (PEI; P3143; Sigma Aldrich) dissolved in a pH = 8.3 borate buffer [14].

Under standard cell culture conditions, dissociated cells were seeded in Modified Eagle s Medium/Nutrient Mixture F-12 Ham (DF12) formulated with sodium bicarbonate, 15 mM HEPES, and no phenol red (D6434; Sigma-Aldrich), and supplemented with 10% FCS. At 2 h after plating, this medium was entirely removed and replaced by the same medium simply supplemented with 20 µg/mL of bovine insulin (I5500; Sigma Aldrich) (DF12i). Subsequently, 70% of the culture medium (i.e., 350/500 µL) was exchanged at day in vitro (div) 1 and 3 and the cultures were generally processed at div7, except when otherwise noted. Note that the cultures also received 1.2 µM of the antimitotic Ara-C [20], 2 h and 18 h after plating, to reach a cumulative concentration of 2.4 µM, which was required to eliminate the vast majority of glial cells from these cultures; under the present conditions, Glial Fibrillary Acidic Protein^+^ astrocytes and CD45^+^ microglial cells usually represent <2% of the total number of cells in the cultures. We have previously shown that the presence of iron in the composition of DF12 promotes low-level oxidative stress-mediated insults causing the progressive loss of DA neurons [15].

We also used a variation of the previous protocol, in which the exposure to DF12i was carried out after an initial period of maturation of the cultures to demonstrate that the effects of test molecules extend beyond a specific time window after plating. Briefly, midbrain cultures were initially maintained in Neurobasal-A medium (#10888022; Thermo Fisher Scientific) supplemented with B27 minus antioxidants (#10889038; Thermo Fisher Scientific), a N2 mix (#17502048; Thermo Fisher Scientific), and 1% FCS. This medium is referred to as a complete Neurobasal medium (cNb). At 2, 18, and 40 h after plating, Ara-C was added to the cultures at a concentration of 1.5 µM to reach a cumulative concentration of 4.5 µM. At the end of div3, the plating medium was completely removed and replaced by an astrocyte-conditioned medium (ACM), prepared as described before [19,21]. At div7, 70% of this medium was replaced by DF12i formulated with phenol red (D6421; Sigma-Aldrich) and supplemented with 20 µg/mL of insulin. MK-801 (2 µM) was also added to the cultures to avoid unwanted excitotoxic stress due to fast washing. This operation was then repeated at div9, 10, and 11 and the cultures were generally processed at div14 for immunocytochemical procedures or DA uptake measurements. DF12 formulated with phenol red was used for mature midbrain cultures, as we observed that it makes iron-mediated neurodegeneration more progressive in this setting.

**Figure 1:**
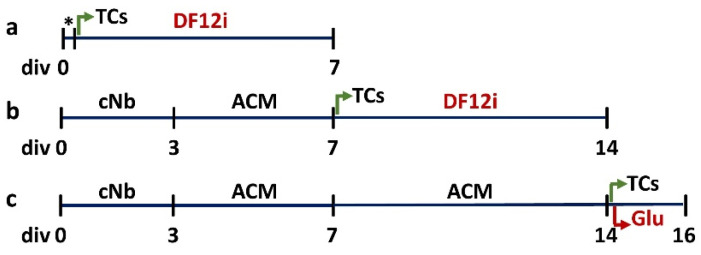
Experimental conditions used to evaluate the neuroprotective potential of TCs for DA neurons in midbrain cultures. (**a**) Standard culture conditions in which DF12i is used to maintain the cultures until div7 after an initial period of 2 h in DF12 medium supplemented with 10% FCS to favor cell attachment (*). (**b**) Culture conditions in which the exposure to DF12i medium between div7–14 is preceded by a period of maturation in cNb and then in ACM. (**c**) Culture conditions in which the initial period in cNb is followed by incubation in ACM until termination of the cultures, at div16. In (**a**,**b**), DA cell death, which is spontaneous, results from the presence of iron in the formulation of DF12i, and in (**c**), from an exposure to exogenous glutamate (Glu).

Finally, to estimate the potential of TCs to block glutamate-mediated DA cell death, we cultivated midbrain cultures as described before, but instead of switching the medium at div7, we kept them in ACM until termination of the experimental protocol. The different protocols are summarized in Figure 1.

### 2.3. Treatments with Test TCs and Reference Compounds

The TC antibiotics, **DOX**, **DMC,** and **CT**, the inhibitor of lipid peroxidation Trolox-C (TROL; #238813), the iron chelator desferrioxamine (DES; D9533), the hydrogen peroxide detoxifying enzyme catalase from bovine liver (CAT; C100), vitamin C (VitC; 255564), and the two non-TC antibiotics erythromycin (ERY; E5389) and streptomycin (STR; S6501) were all from Sigma-Aldrich. The inhibitor of eukaryotic translation, the antifungal antibiotic cycloheximide (CHX; #0970), and the inhibitor of ferroptosis Liproxstatin-1 (LIP; #6113) were from Tocris Biotechne (Abingdon, UK). The two TCs, **DDMC** and **DDOX,** were obtained through in-house synthesis. 

Stock solutions of **DOX** and **CT** were made at 10 mM in distilled water and DMSO, respectively. Stock solutions of other TCs, **DDOX**, **DMC,** and **DDMC,** were prepared at 50 mM in DMSO. All stock solutions were stored at −20 °C. Intermediate dilutions of TCs used for cell culture treatments were made in distilled water and stored for 7 days at 4 °C, protected from light. Stock solutions of TROL and LIP were made at 50 mM in pure ethanol and DMSO, respectively. DES and VitC stock solutions were made at 10 mM in distilled water. CAT was prediluted at 5000 UI in distilled water and kept at 4 °C until use. Treatments with test compounds were carried out or renewed at the time of culture medium change.

### 2.4. Synthesis and Spectrometric Characterization of the Two Novel TCs DDOX and DDMC

**Figure 2:**
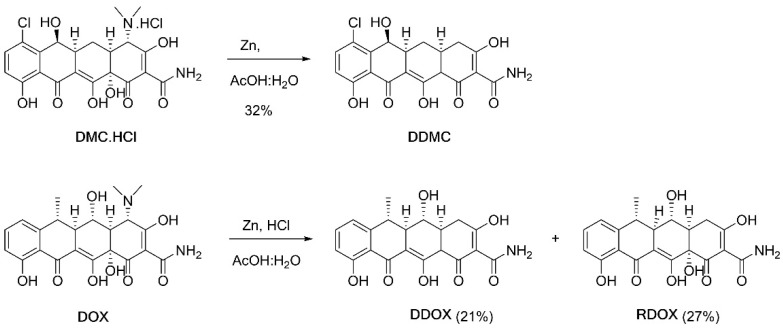
Synthetic route to **DDMC**, **DDOX**, and **RDOX**.

**DDMC** was synthesized from **DMC** HCl by concomitant reduction of the dimethylamino group at position 4 and hydroxy group at position 12a, with the help of zinc dust in an acetic acid/water mixture [22]. **DDOX** was obtained through a similar protocol requiring a reduction step from **DOX**. **RDOX,** generated along with **DDOX**, is the product of mono-reduction of **DOX** where only the dimethylamino function at C_4_ has been reduced (Figure 2).

**DDMC**: In a 50 mL round-bottom flask, **DMC** hydrochloride (300 mg, 0.6 mmol, 1.0 equiv) was suspended in AcOH (6.2 mL) with water (1 mL); then, zinc dust (391 mg, 6.02 mmol, 10 equiv) was added and the reaction mixture was stirred for 20 h at room temperature. The resulting solution was filtered through a small pad of Celite with AcOH. The organic phase was extracted with CH_2_Cl_2_, washed with HCl (1 M) and brine, dried over MgSO_4_, filtered off, and concentrated in vacuo. Precipitation in EtOAc/*n*-pentane produced **DDMC** as a yellow solid (130.5 mg, 32%). Further purification was performed with preparative HPLC (XSelect 4.6 x 150 mm 5 µm, H_2_O + 0.1%TFA: MeCN, 25:75 to 80:20 over 20 min, tr = 11.8 min).

^1^H Nuclear Magnetic Resonance Spectroscopy (NMR) (400 MHz, DMSO-*d*_6_): δ 18.45 (brs, 1H, OH), 14.71 (s, 1H, OH), 11.91 (s, 1H, OH), 9.11 (brs, 1H, NH), 8.72 (s, 1H, NH), 7.59 (d, *J =* 8.9 Hz, 1H), 6.95 (d, *J =* 8.9 Hz, 1H), 5.71–4.88 (brs, 1H, OH), 4.72 (d, *J =* 2.7 Hz, 1H), 3.68 (brs, 1H, CH12a), 3.17 (brs, 1H, OH), 2.82 (ddd, *J =* 11.4, 5.6, 2.7 Hz, 1H), 2.46–2.55 (m, 3H), 1.87 (q, *J =* 12.2 Hz, 1H), 1.70 (ddd, *J =* 12.7, 5.6, 2.4 Hz, 1H) ppm. ^13^C NMR (101 MHz, DMSO): δ 195.32, 191.73, 190.84, 177.76, 173.24, 160.12, 140.88, 136.60, 122.16, 118.72, 116.03, 104.33, 99.19, 63.91, 50.96, 37.97, 36.99, 29.05, 26.65 ppm. High Resolution Mass Spectrometry (HRMS) (ESI) *m/z*: calculated for C_19_H_17_ClNO_7_ [M+H]^+^: 406.0688, found 406.0694.

**DDOX** and **RDOX**: In a 500 mL round-bottom flask, **DOX** monohydrate (5.5 g, 11.9 mmol, 1.0 eq) was suspended in water (50 mL) and stirred, followed by the addition of 35% HCl (1.5 mL, 17 mmol, 1.4 equiv) and AcOH (50 mL). After the dissolution of the mixture, zinc dust was added (7.8 g, 119 mmol, 10 equiv) and the reaction mixture was stirred at room temperature for 4 h. The resulting mixture was filtered through a small pad of Celite with AcOH. The organic phase was extracted with CH_2_Cl_2_, washed with HCl (1 M) and brine, dried over MgSO_4_, filtered off, and concentrated in vacuo. The purification of the residue was performed on an automatic combi flash chromatography (Petroleum ether: Acetone + 1% HCOOH; silicagel column Buchi Flashpure 120 g; 0% to 10% acetone over 20 min, then 10% to 11.3% acetone over 5.1 min, isocratic for 22.7 min, and 11.3 to 20% acetone over 10.4 min (total 58.2 min)). A first fraction (11–18 min) afforded **DDOX** as an amorphous yellow solid (950 mg, 21%), followed by fraction n°2 (19–27 min) as **RDOX** (1310 mg, 27%).

**DDOX**: ^1^H NMR (400 MHz, Acetone-*d*_6_) δ 18.49 (s, 1H), 14.91 (s, 1H), 12.15 (s, 1H), 9.24 (s, 1H), 7.70 (s, 1H), 7.51 (t, *J =* 7.9 Hz, 1H), 6.84 (d, *J =* 7.5 Hz, 1H), 6.76 (d, *J =* 8.3 Hz, 1H), 4.54 (brd, *J =* 6.6 Hz, 1H), 3.94 (d, *J =* 4.9 Hz, 1H), 3.71 (q, *J =* 7.5 Hz, 1H), 3.35 (ddd, *J =* 10.5, 9.0, 5.0 Hz, 1H), 2.81 (d, *J =* 4.6 Hz, 1H), 2.63 (ddd, *J =* 13.5, 8.7, 4.6 Hz, 1H), 2.44–2.31 (m, 2H), 1.48 (d, *J =* 7.4 Hz, 3H). ^13^C NMR (101 MHz, Acetone-*d*_6_) δ 201.06, 197.50, 191.26, 174.77, 168.87, 163.67, 148.75, 138.28, 120.90, 116.20, 115.53, 103.93, 99.86, 70.98, 49.05, 46.74, 39.82, 37.30, 32.34, 22.96. HRMS (ESI) *m/z*: calculated for C_20_H_19_NO_7_ [M+H]^+^ 386.1234, found 386.1238.

**RDOX**: ^1^H NMR (300 MHz, DMSO-*d*_6_): δ 15.36 (s, 1H), 11.53 (s, 1H), 8.85, 8.74 (2brs, each 1H, NH_2_), 7.51 (t, *J =* 8.0 Hz, 1H), 6.91 (d, *J =* 8.0 Hz, 1H), 6.86 (d, *J =* 8.0 Hz, 1H), 6.75 (brs, 1H), 5.25 (brd, *J =* 5.4 Hz, 1H), 3.47 (m, 1H), 2.98–2.75 (m, 2H), 2.60 (p, *J =* 6.7 Hz, 1H), 2.31 (dd, *J =* 12.2, 8.4 Hz, 1H), 2.24 (dm, *J =* 11.3 Hz, 1H), 1.44 (d, *J =* 6.7 Hz, 3H) ppm. ^13^C NMR (75 MHz, DMSO-*d*_6_): δ 194.94, 192.47, 192.02, 176.83, 173.34, 161.06, 148.04, 136.47, 115.80, 115.62, 115.53, 106.62, 98.08, 74.57, 67.64, 62.21, 45.91, 43.04, 29.27, 15.86 ppm. HRMS (ESI) *m/z*: calculated for C_20_H_20_NO_8_ [M+H]^+^: 402.1183, found 402.1189.

**Figure 3:**
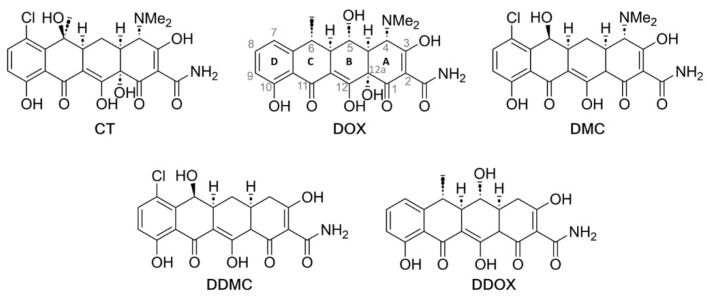
TCs tested in this study. Conventional numbering of the condensed rings and key positions are shown for **DOX**. The four rings of the TC core structure are designated by A,B,C,D.

The chemical structures of all test TCs are given thereafter (Figure 3).

### 2.5. Evaluation of the Antimicrobial Activity of Test TCs

Antibiotic activities of TCs were evaluated against Gram negative, i.e., *P. aeruginosa* (PAO1) and *E. coli* (ATCC 25922), and Gram positive, i.e., *S. aureus* (ATCC 25923) bacterial strains using standardized protocols [23,24]. Table 1 gives an overview of the antibiotic activities of commercially available and newly synthetized TCs. Data expressed in minimal inhibitory concentrations (MICs; µM) confirm that **DOX**, **DMC,** and **CT** possess strong antimicrobial activities against the three bacterial strains tested. Conversely, **DDOX** is devoid of antimicrobial activity whereas **DDMC** shows only marginal activity against *S. aureus*.

### 2.6. Novel Non-Antibiotic TCs Have the Capacity to Penetrate the Brain

Given that **DDOX** and **DDMC** are potential drug candidates for PD, we compared the brain permeability of these two compounds to that of **DOX** used as a reference TC. Specifically, adult Swiss mice receiving a single subcutaneous injection of 40 mg/kg of **DDOX**, **DDMC,** or **DOX** diluted in saline with 5% DMSO and 5% Tween 80 were sacrificed 30 min, 1 h, 8 h, and 24 h after treatment. After sacrifice, brain and serum samples were collected and processed at each time point for TC dosage, using an UHPLC system coupled with a triple quadrupole mass spectrometer LCMS-8030 (Shimadzu Corporation, Kyoto, Japan). The brain-to-plasma ratio calculated from the area under the concentration time curves in the brain and plasma were 0.27 ± 0.13, 0.21 ± 0.05, and 0.11 ± 0.03 (n = 3) for **DDMC**, **DDOX,** and **DOX**, respectively, suggesting that the two novel non-antibiotic TCs penetrate the brain better than **DOX**.

### 2.7. Immunodetection of DA Neurons

After the termination of treatments, midbrain cultures were fixed for 12 min in Dulbecco’s phosphate-buffered saline (PBS) containing 4% formaldehyde (#252549; Sigma Aldrich), washed with PBS and then incubated for 18 h with a mouse anti-tyrosine hydroxylase (TH) antibody (#22941; ImmunoStar; 1:1000 in 0.2% Triton X-100/PBS). After recovery of the antibody, which is reusable several times, the cultures were exposed to an anti-rabbit IgG (H+L) conjugated to Alexa Fluor 555. Images of TH^+^ neurons are presented in an inverted form for better visualization of neuronal morphology.

### 2.8. Cell Counting Operations

For cell counting operations, we used a Nikon Eclipse Ti-U fluorescence inverted microscope (Nikon France, Champigny sur Marne, France) equipped with an ORCA-Flash digital camera (Hamamatsu Photonics, Massy, France) and the NIS-Elements software Version 5.41 (Nikon). The number of TH^+^ neurons was estimated by visually inspecting with a 10× objective 10–15 visual fields that were randomly selected for each treatment condition.

### 2.9. Assessment of Concomitant Changes in ROS Levels and Mitochondrial Membrane Potential

After being removed, the culture medium from each individual culture well was transferred to another culture plate that was placed in a CO_2_ incubator at 37 °C for temporary storage. This medium was immediately replaced by warm PBS-glucose (5 mM) and the cultures were then exposed for 35 min to tetramethylrhodamine methyl ester, perchlorate (TMRM; 50 nM; ab228569; Abcam, Cambridge, UK) together with dihydrorhodamine 123 (DHR-123; 25 µM; D23806; Thermo Fisher Scientific) 10 min later [15,25] to assess, concomitantly, changes in mitochondrial membrane potential (ΔΨm) and intracellular ROS levels, respectively. The cultures were then washed extensively (3×) with PBS-glucose to remove the fluorescent probes in excess and the cultures were then re-incubated with the culture medium stored in the incubator.

In some experiments, ROS production was induced with H_2_O_2_ (25 µM; #8070.4; CARL ROTH, Karlsruhe, Germany) and mitochondrial depolarization with the protonophore carbonyl cyanide 4-(trifluoromethoxy)phenylhydrazone (FCCP; 0.5µM) provided in the TMRM assay kit, using APO (100µg/mL)-treated cultures to avoid spontaneous degenerative changes. H_2_O_2_ and FCCP were added 1 h and 10 min before addition of the fluorogenic probes, respectively. Note that FCCP but not H_2_O_2_ remained present during the whole incubation period in PBS-glucose.

For each culture condition, fluorescent images from at least five randomly chosen fields were acquired with a 40× fluorescence objective using a Nikon Eclipse Ti-U fluorescence inverted microscope equipped with an ORCA Flash digital camera. The excitation and emission wavelengths for DHR-123 were 490 nm and 525 nm, respectively, whereas the corresponding values for TMRM were 548 nm and 575 nm, respectively. Results were expressed in changes of fluorescence intensity relative to APO-treated cultures. The open-source FIJI software [26] was used for quantification of the fluorescent signals.

### 2.10. Tritiated-DA Uptake

The uptake of tritiated-DA was performed to evaluate the functional integrity and synaptic function of DA neurons as described before [15,19]. Briefly, after culture medium removal, midbrain cultures were incubated at 37 °C in PBS-glucose (5 mM) containing 25 nM [^3^H]-DA (NET673; 40 Ci/mmol; PerkinElmer, Courtaboeuf, France). After 15 min, the supernatant containing the radiochemical in excess was removed and cultured cells were washed twice with PBS-glucose before a lysis step with 1% Triton X-100 in distilled water. Cell lysates were then recovered in 2 mL of Econofluor-2 (#6NE9699; PerkinElmer) and the radioactivity accumulated by DA neurons quantified by liquid scintillation spectrometry using a Tri-Carb 4910TR counter (PerkinElmer). Blank values were obtained by incubating APO-treated cultures with 0.2µM GBR-12909 (Sigma-Aldrich).

### 2.11. Statistical Analysis

For statistical analyses, we used One-way ANOVA followed by the post hoc Dunnett’s test for all comparisons against a control group and the Student–Newman–Keuls test (SNK) post hoc test for all pairwise comparisons.

## 3. Results

### 3.1. The TC Antibiotics DOX and DMC Prevent Iron-Dependent DA Cell Death in Midbrain Cultures

**Figure 4:**
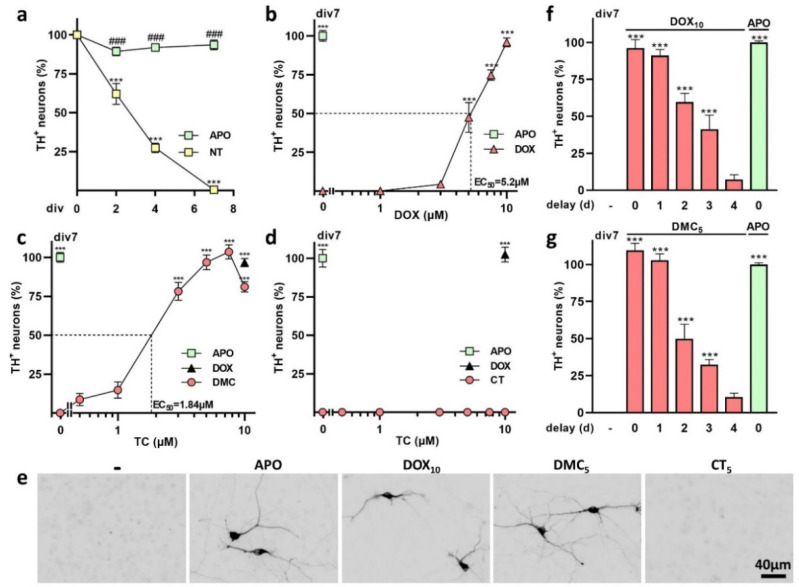
Protection of cultured DA neurons by **DOX** and **DMC**. (**a**) Kinetics of spontaneous DA (TH^+^) cell death in midbrain cultures maintained in DF12i medium after plating until div7. APO (100 µg/mL) provides robust neuroprotection under these conditions. Data expressed in % of DA neurons at div0 are means ± SEM (8–12). *** *p* < 0.001 vs. NT, at div0; ^###^ *p* < 0.001 vs. NT of same culture age. One-way ANOVA followed by SNK’s test. (**b**–**d**) Survival of TH^+^ neurons in midbrain cultures chronically exposed to different concentrations (0.5–10 µM) of **DOX**, **DMC,** or **CT** until div7. APO (100 µg/mL) is used as a reference neuroprotective treatment in (**b**–**d**). **DOX** (10 µM) is used as a reference TC in (**c**,**d**). Data expressed in % of APO-treated cultures in (**b**–**d**) are means ± SEM (4–9). *** *p* < 0.001 vs. NT. One-way ANOVA followed by Dunnett’s test. (**e**) Representative photomicrographs of TH^+^ neurons in div7 midbrain cultures chronically exposed or not to **DOX** (10 µM), **DMC** (5 µM), **CT** (5 µM), or APO (100 µg/mL). (**f**,**g**) Survival of DA neurons at div7, under conditions where treatments with **DOX** (10 µM) or **DMC** (5 µM) are carried out with or without a time delay (d) after plating. Data expressed in % of APO-treated cultures are means ± SEM (4–14). *** *p* < 0.001 vs. NT. One-way ANOVA followed by Dunnett’s test.

To study the neuroprotective mechanism of action of **DOX** and other TCs for DA neurons, we established an experimental model of midbrain cell cultures in which DA cell loss develops progressively and spontaneously as a function of time in DF12i medium (Figure 1 and Figure 4a). The spontaneous death of DA neurons results from (i) the presence of trace levels (1.6 µM) of iron in DF12 medium used for the maintenance of the cultures and (ii) the elimination of astrocytes from the cultures by treatment with the antimitotic Ara-C [15]. As a result, the iron-chelating glycoprotein APO (100 µg/mL) efficiently protects DA neurons from degenerating in this experimental setting (Figure 4a).

Figure 4b,c depicts the survival-promoting effects of **DOX** and **DMC** (0.5–10 µM) for DA neurons in div7 midbrain cultures, i.e., a stage where DA cell loss is virtually complete in non-treated (NT) cultures. We found that **DOX** has a strong neuroprotective potential for DA neurons in this setting. Specifically, **DOX** exerted partial rescue effects at 5 µM and optimal protection at 10 µM. At a concentration of 2.5 µM, however, **DOX** had virtually no protective effect. The effective concentration 50 (EC_50_) of **DOX** protecting half of DA neurons was estimated graphically at 5.2 µM in this paradigm. **DMC** appeared more potent than **DOX,** as significant protective effects were already observed at concentrations as low as 1 µM, with the EC_50_ of this compound being estimated at 1.84 µM. **CT**, which differs from **DMC** only by a methyl group located at position C_6_ on ring C of the TC core structure, provided no significant protection to DA neurons regardless of the test concentration (Figure 4d). APO (100 µg/mL) is used as a reference protective treatment in Figure 4b–d and **DOX** (10 µM) as a reference TC in Figure 4c,d. Figure 4e illustrates the impact that treatments with **DOX** (10 µM), **DMC** (5 µM), **CT** (5 µM), and APO (100 µg/mL) exert on DA (TH^+^) neurons in div7 midbrain cultures chronically exposed to these treatments.

Interestingly, **DOX** and **DMC** were both found to exert protective effects when applied with delay to the cultures (Figure 4f,g). Experimental data show that approximately half of DA neurons were still rescued at div7 when treatments with **DOX** and **DMC** were applied at div2, i.e., at a stage where the neurodegenerative process is already engaged but partial. If the treatment with **DOX** or **DMC** was initiated at div3, approximately 30% of DA neurons still survived at div7. Starting the treatments at div4 and onwards resulted in limited or no protection at all. **CT** was ineffective in this paradigm (not shown).

### 3.2. Protective Effects of TC Antibiotics Are Not Reproduced by Other Antimicrobial Molecules

**Figure 5:**
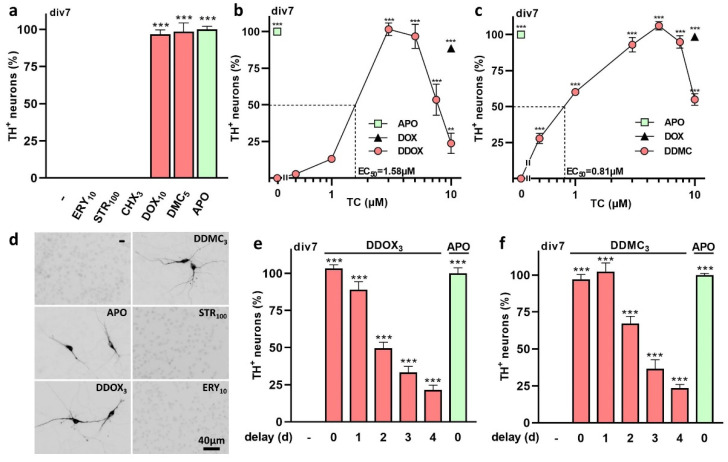
Protection of cultured DA neurons by non-antibiotic TCs. (**a**) Survival of TH^+^ neurons in midbrain cultures chronically treated with ERY (10 µM), STR (100 µM), CHX (3 µM), **DOX** (10 µM), **DMC** (5 µM), or APO (100 µg/mL) until div7. Data expressed in % of APO-treated cultures are means ± SEM (3–9). *** *p* < 0.001 vs. NT. One-way ANOVA followed by Dunnett’s test. (**b**) Survival of TH^+^ neurons in midbrain cultures chronically treated with **DDOX** (0.5–10 µM), **DOX** (10 µM), or APO (100 µg/mL) until div7. (**c**) Survival of TH^+^ neurons in midbrain cultures chronically treated with **DDMC** (0.5–10 µM), **DOX** (10 µM), or APO (100 µg/mL) until div7. Data in (**b**,**c**) expressed in % of APO-treated cultures are means ± SEM (4–12). *** *p* < 0.001 vs. NT. One-way ANOVA followed by Dunnett’s test. (**d**) Representative photomicrographs of TH^+^ neurons in div7 midbrain cultures exposed or not exposed to **DOX** (10 µM), **DDOX** (3 µM), **DDMC** (3 µM), ERY (10 µM), STREP (100 µM), or APO (100 µg/mL). (**e**,**f**) Survival of DA neurons at div7, under conditions where treatments with **DDOX** (3 µM) or **DDMC** (3 µM) are carried out with or without a time delay (d) after plating. Comparison with a chronic treatment with APO. Data expressed in % of APO-treated cultures are means ± SEM (5–8). *** *p* < 0.001 vs. NT. One-way ANOVA followed by Dunnett’s test.

We tested whether two other unrelated antimicrobial molecules, such as the macrolide ERY or the β-lactam STR, could also protect DA neurons from iron-mediated neurodegeneration. We found that antimicrobial concentrations of ERY (10 µM) or STR (100 µM) [27] that are not toxic for neuronal cells could not mimic the rescue effects of **DOX** or **DMC** for DA neurons (Figure 5a). The antifungal antibiotic CHX [28] was similarly inactive, leading us to assume that neuroprotection by **DOX** and **DMC** is unrelated to the antibiotic activity of these compounds (Figure 5a).

### 3.3. Non-Antibiotic TC Derivatives Provide Robust Neuroprotection for DA Neurons

To further study the mechanisms underlying TC-mediated neuroprotection, we evaluated the protective potential of newly designed TC analogs having limited or no antibiotic activity (see Table 1). The two new molecules, **DDOX** and **DDMC**, which are doubly reduced derivatives of **DOX** and **DMC**, respectively, were both highly effective in protecting DA neurons (Figure 5b,c). 

The EC_50s_ for **DDOX** and **DDMC** were estimated at 1.58 and 0.81 µM, respectively. Figure 5d illustrates the impact that treatments with **DDOX** (3 µM), **DDMC** (3 µM), ERY (10 µM), STR (100 µM), and APO (100 µg/mL) exert on DA neurons in div7 midbrain cultures chronically exposed to these treatments. If **DDOX** (3 µM) or **DDMC** (3 µM) were added to the cultures with a delay, the rescue of DA neurons by these two compounds was significant if the treatment was not postponed by more than 4 d (Figure 5e,f).

### 3.4. Protection by DOX and Other Protective TCs against Spontaneous DA Cell Death Does Not Occur through Inhibition of Excitotoxic Stress

**Figure 6:**
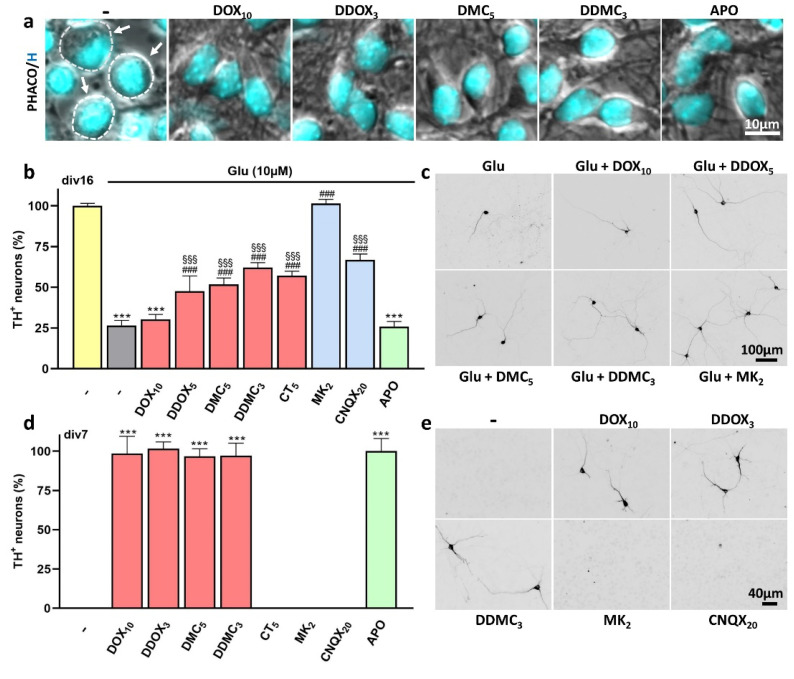
TC-mediated neuroprotection of DA neurons against iron-mediated neurodegeneration is not explained by blockade of glutamate excitotoxicity. (**a**) Phase contrast (Phaco) images of div2–3 cultures showing that protective TC derivatives and APO prevent the swelling of neuronal cell bodies that is prominent when iron-dependent insults culminate in NT cultures. White arrows point to swollen neuronal cell bodies whose boundaries are delineated with a dashed line. Note that nuclear integrity (H-33342; blue signal) is preserved not only in the presence, but also in the absence, of protective treatments at this stage of neurodegeneration. (**b**) Survival of div16 DA neurons exposed 2 d before to Glu (10 µM) in the presence or absence of **DOX** (10 µM), **DDOX** (5 µM), **DMC** (5 µM), **DDMC** (3 µM), **CT** (5 µM), APO (100 µg/mL), MK-801 (MK, 2µM), or CNQX (20 µM). Data expressed in % of NT cultures are means ± SEM (6–15). *** *p* < 0.001 vs. NT; ^###^ *p* < 0.001 vs. Glu; ^§§§^ *p* < 0.001 vs. MK. One-way ANOVA followed by SNK’s test. (**c**) Photomicrographs illustrating the survival of TH^+^ neurons in div16 midbrain cultures exposed 2 d before to Glu (10 µM) in the presence or absence of some of the treatments described in (**b**). (**d**) Survival of div7 DA neurons maintained in DF12i in the presence or absence of **DOX** (10 µM), **DDOX** (3 µM), **DMC** (5 µM), **DDMC** (3 µM), APO (100 µg/mL), MK-801 (MK, 2 µM), or CNQX (20 µM). Data expressed in % of APO-treated cultures are means ± SEM (3–11). *** *p* < 0.001 vs. NT. One-way ANOVA followed by Dunnett’s test. (**e**) Photomicrographs illustrating the survival of TH^+^ neurons in div7 midbrain cultures exposed to some of the treatments described in (**d**).

We noted that neuroprotective TCs prevent the swelling of neuronal cell bodies when iron-dependent insults culminate in NT cultures (Figure 6a). With cell body swelling being classically observed in situations where ionotropic glutamate receptor over-activation leads to excitotoxic neuronal death [29,30], we assessed the protective potential of TCs in a situation where DA neurons are exposed to 10 µM glutamate to promote low-level excitotoxic insults. We found that all TC derivatives except **DOX** substantially blocked glutamate-mediated DA cell death (Figure 6b,c). As expected, the blockade of NMDA receptors by MK-801 (2 µM) or AMPA/kainate receptors by CNQX (20 µM) provided robust and intermediary protection, respectively, under the same experimental conditions [31]. However, neither MK-801 (2 µM) nor CNQX (20 µM) mimicked TC-mediated neuroprotection when DA neurons were subjected to iron-mediated insults (Figure 6d,e).

### 3.5. Neuroprotective TCs Prevent Intracellular ROS Production and Preserve Mitochondrial Health

**Figure 7:**
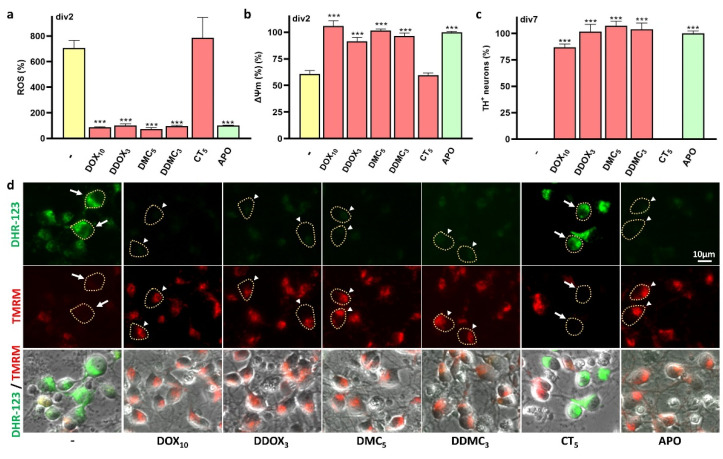
**DOX**, **DDOX**, **DMC,** and **DDMC** limit oxidative stress and preserve mitochondrial membrane potential (ΔΨm). (**a**,**b**) ROS production and changes in ΔΨm monitored with the fluorogenic probes DHR-123 and TMRM, respectively, in div2 midbrain neurons exposed or not exposed to **DOX** (10 µM), **DDOX** (3 µM), **DMC** (5 µM), **DDMC** (3 µM), or **CT** (5 µM). APO (100 µg/mL) was used as a reference neuroprotective treatment. Data expressed in % of APO-treated cultures are means ± SEM (4–12). *** *p* < 0.001 vs. NT. One-way ANOVA followed by Dunnett’s test. (**c**) Survival-promoting effects of treatments described in (**a**,**b**). Data expressed in % of APO-treated cultures are means ± SEM (4–16). ***p < 0.001 vs. NT. One-way ANOVA followed by Dunnett’s test. (**d**) Images showing DHR-123 (upper panel) and TMRM (mid panel) fluorescent signals in div2 midbrain cultures treated or not treated with **DOX**, **DDOX**, **DMC**, **DDMC,** or **CT**. The lower panel represents Phaco images merged with the DHR-123 and TMRM fluorescent signals. White arrows point to neurons showing a decrease in ΔΨm with an increase in ROS production. White arrowheads indicate neurons, in which ΔΨm is preserved and ROS production contained. Yellow dotted lines represent virtual boundaries of neuronal cell bodies indicated by white arrows or white arrowheads.

To better understand the nature of the protective mechanism of TCs, we used the fluorogenic probe DHR-123 for monitoring intracellular ROS production in TC-treated and NT cultures. We found that ROS levels increased progressively after div2–3 if APO was omitted from the culture medium. When APO was replaced by **DOX** (10 µM), **DDOX** (3 µM), **DMC** (5 µM), or **DDMC** (3 µM), ROS returned to basal levels estimated in the presence of APO (Figure 7a). The TC antibiotic **CT** (5 µM) could not prevent ROS production.

Concomitant to ROS quantification with DHR-123, we also monitored changes in ΔΨm with the mitoprobe TMRM. We established that neuroprotective TCs but not **CT** prevented the decrease in ΔΨm occurring in ROS-producing neurons in NT cultures (Figure 7b). ΔΨm was also well preserved in APO-treated cultures. Figure 7c describes the impact that chronic treatments with TCs and APO have on the survival of TH^+^ neurons in div7 midbrain cultures. Photomicrographs from Figure 7d illustrate the impact of treatments with TCs and APO on ROS production (upper row; DHR-123) and ΔΨm (middle row; TMRM) in div2 midbrain cultures. The bottom row represents phase contrast (Phaco) images merged with DHR-123 and TMRM fluorescent signals.

### 3.6. TCs Prevent Iron-Catalyzed Oxidative Stress and Its Consequences

**Figure 8:**
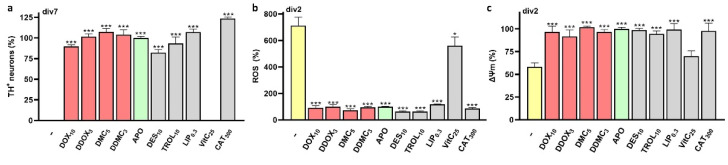
**DOX**, **DDOX**, **DMC,** and **DDMC** protect DA neurons by inhibiting oxidative insults elicited through a Fenton-type reaction. (**a**) Survival of TH^+^ neurons in div7 midbrain cultures chronically exposed or not exposed to **DOX** (10 µM), **DDOX** (3 µM), **DMC** (5 µM), **DDMC** (3 µM), the iron chelator desferrioxamine (DES; 10 µM), the inhibitor of lipid peroxidation TROL (10 µM), the inhibitor of ferroptosis LIP (0.3 µM), the H_2_O_2_ detoxifying enzyme CAT (300 UI/mL), the water-soluble vitamin VitC (25 µM), and APO (100 µg/mL). Data expressed in % of APO-treated cultures are means ± SEM (3–18). *** *p* < 0.001 vs. NT. One-way ANOVA followed by Dunnett’s test. (**b**) ROS production monitored with the fluorogenic probe DHR-123 in div2 midbrain neurons exposed or not exposed to the same treatments as in (**a**). (**c**) Changes in ΔΨm measured in the same cultures as in (**a**) using the mitoprobe TMRM. Data in (**b**,**c**) expressed in % of APO-treated cultures are means ± SEM (3–7). * *p* < 0.05, *** *p* < 0.001 vs. NT. One-way ANOVA followed by Dunnett’s test.

To better understand the nature of the neuroprotective effects of TCs, we performed a series of experiments comparing the neuroprotective action of TCs to that of other treatments of interest. These treatments include the iron chelator DES (10 µM), the inhibitors of lipid peroxidation and ferroptosis TROL (10 µM) and LIP (0.3 µM), respectively, the H_2_O_2_ neutralizing enzyme CAT (300 IU/mL), the water-soluble vitamin VitC (25 µM), and APO (100 µg/mL). Results are expressed in % of APO-treated cultures. We showed that DES, TROL, LIP, and CAT promote the survival of DA neurons at div7 with an efficacy comparable to that of neuroprotective TCs and APO. VitC, however, had no protective effect for DA neurons in this setting (Figure 8a).

We then monitored ROS production and changes in ΔΨm in div2 cultures receiving the same treatments as before (Figure 8b,c). As expected, intracellular oxidative stress significantly increased in div2 midbrain cultures receiving no treatment, whereas a decrease of ΔΨm was observed, concomitantly. Conversely, ROS levels and ΔΨm returned to basal levels in the presence of APO and non-TC compounds with an antioxidant potential except for VitC, which only marginally reduced ROS production and failed to maintain the ΔΨm. **DOX**, **DDOX**, **DMC,** and **DDMC** prevented ROS production and preserved ΔΨm under these conditions, as expected.

### 3.7. Neuroprotective TCs Are Inoperative against Oxidative Insults or Mitochondrial Deficits Generated Acutely

**Figure 9:**
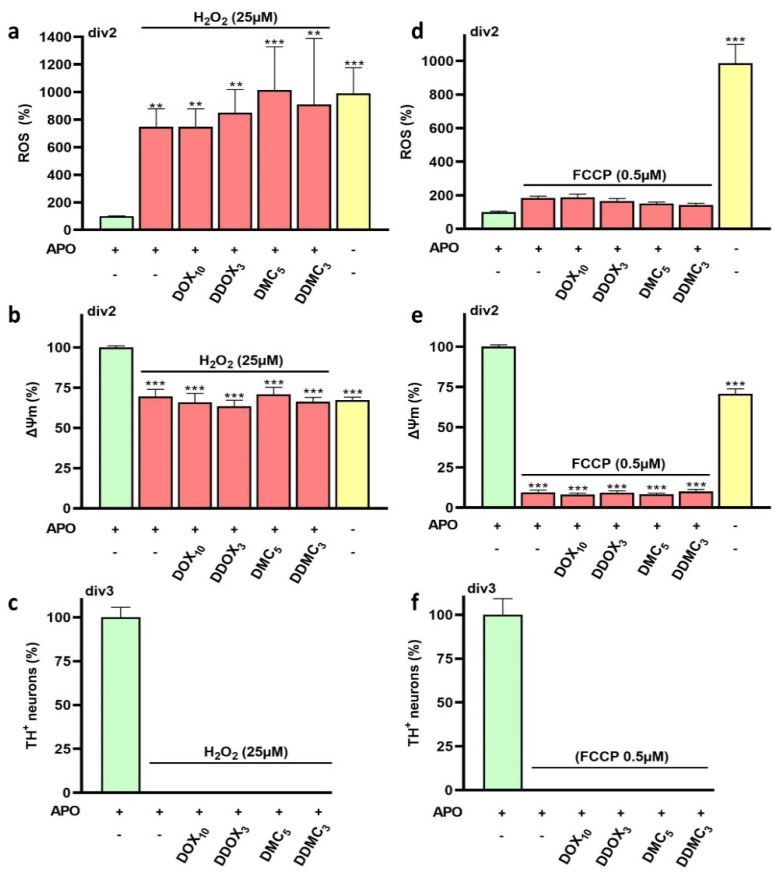
**DOX**, **DDOX**, **DMC,** and **DDMC** are ineffective against acute oxidative insults or mitochondrial deficits induced in APO-treated cultures. (**a**,**b**) ROS and ΔΨm monitored with DHR-123 and TMRM, respectively, in div2 midbrain cultures treated with APO (100 µg/mL) to prevent spontaneous DA cell loss and challenged acutely with H_2_O_2_ (25 µM) in the presence or absence of **DOX** (10 µM), **DDOX** (3 µM), **DMC** (5 µM), or **DDMC** (3 µM). As a comparison, we also estimated ROS levels in div2 sister cultures not treated with APO. Data expressed in % of APO-treated cultures are means ± SEM (5–10). ** *p* < 0.01, *** *p* < 0.001 vs. APO, only. One-way ANOVA followed by Dunnett’s test. (**c**) Survival of DA neurons in APO-treated midbrain cultures challenged acutely at div2 with H_2_O_2_ in the presence or absence of the test TCs and then left to recover with the same treatments until div3 for survival assessment. Data expressed in % of APO-treated cultures are means ± SEM (n = 3). (**d**,**e**) ROS and ΔΨm production monitored in div2 midbrain cultures treated with APO and challenged acutely with FCCP (0.5 µM) in the presence or absence of the same treatments as in (**a**). As a comparison, ROS levels and changes in ΔΨm were estimated in div2 cultures not treated with APO. Data expressed in % of APO-treated cultures are means ± SEM (5–10). *** *p* < 0.001 vs. APO, only. One-way ANOVA followed by Dunnett’s test. (**f**) Survival of DA neurons in APO-treated midbrain cultures challenged acutely at div2 with FCCP (0.5 µM) in the presence or absence of the test TCs and then left to recover with the same treatments until div3 for survival assessment. Data expressed in % of APO-treated cultures are means ± SEM (n = 3).

In a separate set of experiments described in Figure 9, we tested whether **DOX**, **DDOX**, **DMC,** and **DDMC** could limit acute oxidative or mitochondrial insults generated by a transient exposure to the oxidizing agent H_2_O_2_ (25 µM) or the protonophore FCCP (0.5 µM), which operates as an uncoupler of oxidative phosphorylation [32].

Using div2 midbrain cultures treated with APO to preclude spontaneous neurodegenerative events, we established that neither **DOX**, **DDOX**, **DMC,** nor **DDMC** could restrain oxidative stress-mediated insults when APO-treated cultures are challenged with 25 µM H_2_O_2_ for 1 h (Figure 9a). None of these TCs could prevent the concomitant decrease of ΔΨm associated with ROS production under these conditions (Figure 9b). In line with these observations, DA neurons were not protected when acutely challenged with H_2_O_2_ in the presence of **DOX**, **DDOX**, **DMC,** or **DDMC** and then left to recover in the presence of the same TCs until div3 for survival assessment (Figure 9c). Note that changes in ROS levels and ΔΨm in div2 cultures acutely challenged with H_2_O_2_ were relatively similar to those observed in sister cultures where neurodegenerative changes occur spontaneously (Figure 9a,b).

**DOX**, **DDOX**, **DMC,** and **DDMC** also failed to prevent the decrease in ΔΨm observed in APO-treated cultures acutely exposed to FCCP (0.5 µM) (Figure 9d,e). There were, however, no significant changes in ROS production under FCCP treatment, while test TCs did not modify ROS levels under these conditions. We also found that DA neurons were not protected when acutely challenged with FCCP in the presence of **DOX**, **DDOX**, **DMC,** or **DDMC** and then left to recover in the presence of the same TCs until div3 for survival assessment (Figure 9f). We observed the expected changes in ROS levels and ΔΨm in div2 sister cultures not treated with APO.

Overall, these observations suggest that neither **DOX**, **DDOX**, **DDMC,** nor **DMC** are effective against acute oxidative or mitochondrial insults induced in APO-treated cultures.

### 3.8. The Protective Effects of TCs Are Still Observable When Low-Level Iron-Mediated Oxidative Stress Takes Place in Mature Midbrain Cultures

**Figure 10:**
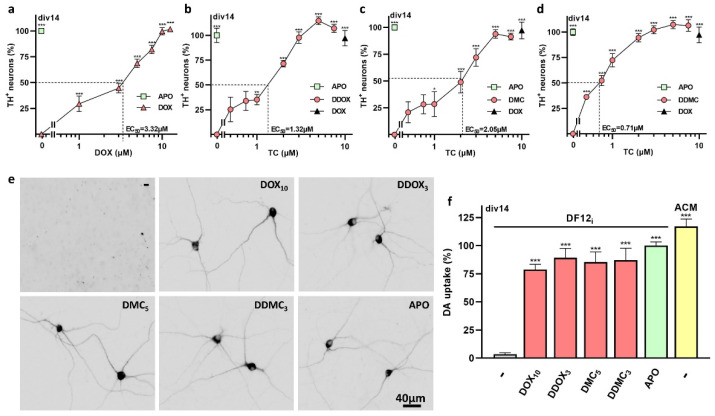
TC derivatives preserve the survival and function of mature DA neurons enduring low-level iron-mediated neurodegeneration. (**a**–**d**) Concentration–response curves of the rescue effects of **DOX**, **DDOX**, **DMC,** and **DDMC** for DA neurons, in div14 midbrain cultures, which have been gradually placed since div7 in DF12i supplemented with 2 µM MK-801. APO (100 µg/mL) was used as a reference neuroprotectant. EC_50s_ for each TC are indicated on the graph above. Data expressed in % of APO-treated cultures are means ± SEM (4–9). * *p* < 0.05, ** *p* < 0.01, *** *p* < 0.001 vs. NT. One-way ANOVA followed by Dunnett’s test. (**e**) Microphotographs illustrating the protective effects of **DOX** (10 µM), **DDOX** (3 µM), **DMC** (5 µM), **DDMC** (3 µM), or APO (100 µg/mL) in mature midbrain cultures. (**f**) DA uptake in div14 midbrain cultures that were gradually placed since div7 in DF12i supplemented with 2 µM MK-801 in the presence or absence of **DOX** (10 µM), **DDOX** (3 µM), **DMC** (5 µM), **DDMC** (3 µM), or APO (100 µg/mL). As a comparison, the uptake was also performed in cultures maintained in ACM during the entire culture time. Data expressed in % of APO-treated cultures are means ± SEM (8–18). *** *p* < 0.001 vs. NT. One-way ANOVA followed by Dunnett’s test.

We wanted to confirm that the effects of TCs for DA neurons remain observable in mature midbrain cultures. Precisely, we gradually replaced ACM from div7 midbrain cultures by DF12i supplemented with 2 µM MK-801. The cultures were then maintained in the presence or absence of various concentrations of **DOX**, **DDOX**, **DMC**, or **DDMC** until processing at div14. We used APO (100 µg/mL) as a reference protective treatment and **DOX** (10 µM) as a reference TC molecule when needed. Experimental results clearly show that all TCs remained protective for older DA neurons (Figure 10a–d). Note, however, that **DOX** appeared more potent than under standard experimental conditions. Based on estimated EC_50s_, the potency of test TCs to rescue mature DA neurons appears to display the following order: **DDMC** > **DDOX** > **DMC** > **DOX**. Neuroprotective effects of TCs are illustrated by photomicrographs of TH^+^ neurons in Figure 10e. Note that protective TCs also well preserved the morphology of DA neurons in this experimental setting.

In addition, we measured the uptake of tritiated DA in mature midbrain cultures exposed to optimal concentrations of **DOX** (10 µM), **DDOX** (3 µM), **DMC** (5 µM), or **DDMC** (3 µM). As a comparison, we performed the uptake in cultures maintained throughout the culture time with ACM. Our results show that the uptake of DA is comparable in APO- and TC-treated cultures, suggesting that rescued neurons are fully functional (Figure 10f).

## 4. Discussion

In the present experimental work, we demonstrate that two TC class antibiotics, **DOX** and **DMC,** provide robust and sustained neuroprotection to midbrain DA neurons in culture enduring sustained low-level iron-mediated insults. Non-TC antimicrobial molecules did not have the capacity to protect DA neurons, suggesting that neuroprotection was unrelated to the antibiotic activity of test TCs. Comforting this view, non-antibiotic TC derivatives of **DOX** and **DMC**, namely **DDOX** and **DDMC**, respectively, proved to be robustly protective for DA neurons. Neuroprotection of DA neurons by TCs remained observable regardless of the degree of maturation of midbrain cultures. **DOX**, **DDOX**, **DMC,** and **DDMC** operated similarly by restraining intracellular oxidative stress and preventing mitochondrial dysfunction, i.e., cellular perturbations arising as consequences of iron-mediated ferroptosis. Neuroprotection by test TCs was lost, however, if DA neurons were subjected to acute oxidative or mitochondrial insults.

### 4.1. DOX and DMC, but Not CT, Exert Robust Protective Effects for DA Neurons Enduring Low-Level Intensity Iron-Mediated Insults

Many experimental data suggest that **DOX** and related TCs might have potential therapeutic utility for PD treatment [10,11,12,13,33]. This might be through the inhibition of glial inflammatory processes that contribute to neurodegeneration [10,14,34,35] or the blockade of intrinsic neuronal DA cell death processes such as αS-mediated neurotoxicity [12,13].

Here, we were more specifically interested in investigating whether **DOX** and other TC derivatives could interfere with oxidative stress-mediated insults that represent a common pathogenic mechanism in PD neurodegeneration [5,8,36]. Thus, we established a culture model of midbrain DA neurons in which sustained low-level oxidative stress, mediated by iron, results in the progressive demise of these neurons [15,37]. This model system might be particularly pertinent as iron deposition is a typical feature of PD degeneration [8,17,18]. In this setting, we demonstrate that **DOX** exerts robust protective effects that are concentration-dependent with an EC_50_ of approximately 5 µM. Interestingly, **DMC**, another TC antibiotic reported recently to protect against αS-induced toxicity [38], was even more potent than **DOX** in protecting DA neurons in the same paradigm. Quite unexpectedly, however, **CT**, a TC which differs from **DMC** by a methyl located at the C_6_ position of the tetracyclic core structure [39], failed to afford protection to DA neurons. This suggests that the presence of the C_6_ methyl group in **CT** causes disruption of steric and electronic features presumably essential for neuroprotection by **DMC** in this paradigm of neurodegeneration.

### 4.2. Protective Effects of DOX and DMC Are Not Related to Their Antibiotic Activity

TCs and many other antimicrobial compounds operate by inhibiting bacterial protein translation in particular by targeting the ribosomal machinery [40,41]. Interestingly, some studies report that some of these compounds, including TCs, may also inhibit eukaryotic ribosomes [42], indicating that TC-mediated neuroprotection may be possibly related to the inhibition of protein synthesis in our model system. In support of this view, the TC minocycline was reported to extend the lifespan of both young and old *C. elegans* nematodes by attenuating cytoplasmic mRNA translation [43], and a well-known inhibitor of eukaryotic translation, the antifungal antibiotic compound CHX [28], was found to prevent neuronal programmed cell death induced by nerve growth factor deprivation [44].

Thus, we tested the possibility that non-TC antibiotics, such as the macrolide ERY or the β-lactam STR that both target ribosomal translation, could mimic the neuroprotective effects of **DOX** and **DMC** against iron-mediated DA cell degeneration. At antibiotic concentrations [27], neither ERY nor STR was able to do so, suggesting that the protective effects of TCs are probably unrelated to their capacity to inhibit protein synthesis. Two additional arguments comfort this view: (i) **CT**, which possesses a good antibiotic activity at test concentrations, was not protective in our model system, and (ii) at concentrations known to inhibit eukaryotic translation [28,44], CHX was similarly ineffective.

### 4.3. Non-Antibiotic TC Derivatives Are Strongly Neuroprotective for DA Neurons

A possible long-lasting use of TC antibiotics for PD treatment would probably cause the selection of antibiotic resistance genes in pathogens as well as persistent changes in the commensal host microbiome [45]. Therefore, we designed novel TC analogs having limited or no antibiotic activity using a synthesis strategy inspired by Golub et al. [46]. Indeed, these authors established that the dimethylamino group at C_4_ on the upper half of the TC core structure is essential for the antimicrobial activity of this class of compounds. Concretely, we removed the dimethylamino substituent at position 4 along with the hydroxyl group at position 12a on ring A of the two parent TCs, **DOX** and **DMC**. The new doubly reduced molecules, named **DDOX** and **DDMC**, respectively, were strongly neuroprotective despite having no or little antimicrobial activity, respectively, further comforting the idea that the protective effects of TC derivatives for DA neurons are unrelated to their antimicrobial activity.

### 4.4. A Delayed Treatment with TCs Provides Protection as Long as Neurodegeneration Is Ongoing

We also established that all neuroprotective TCs retain some efficacy to protect DA neurons, as long as the neurodegenerative process is still in progress. More specifically, experimental data show that approximately half of DA neurons could still be rescued at div7 when the treatment with TCs was applied at div2, i.e., at a stage where the neurodegenerative process is already engaged but where a substantial number of DA neurons are still alive. When TCs were applied at div3, DA cell rescue at div7 was still substantial, whereas neuroprotection progressively diminished thereafter. These results are of interest considering that TCs could have potential utility for PD treatment. Indeed, clinical symptoms develop in this disorder when DA cell loss is already substantial [47,48].

### 4.5. TCs Do Not Operate by Preventing Glutamate-Induced Excitotoxicity

Neuroprotective TCs also efficiently prevented the swelling of neuronal cell bodies occurring when degenerative changes culminate in control culture conditions. Neuronal cell body swelling is a typical feature of glutamate-mediated excitotoxicity [29,49]. Therefore, we hypothesized that protective TCs could possibly operate by restraining excitotoxic insults mediated by ionotropic glutamate receptors. Related to this, **DMC** was reported to limit glutamate-induced neurodegeneration [50]. In good agreement with this observation, we found that **DMC** was protective for DA neurons under experimental conditions where these neurons endure low-level excitotoxic insults mediated by glutamate. Interestingly, **DDOX** and **DDMC** were also similarly protective. Several arguments suggest, however, that TCs do not operate by blocking glutamate-mediated neurodegeneration in our paradigm of iron-mediated DA cell loss: (i) **DOX** was protective against iron-mediated DA cell loss, but ineffective against glutamate-mediated neurodegeneration in agreement with previous observations [51]; (ii) Conversely, **CT**, which was protective against glutamate-mediated excitotoxic stress, was ineffective against iron-mediated neurodegeneration; (iii) Most importantly, the protective effects of TCs against iron-mediated insults were not reproduced by blockers of NMDA or AMPA/kainate receptors. Overall, the present data indicate that TCs do not block iron-mediated DA cell death by interfering with a death modality requiring activation of ionotropic glutamate receptors. Therefore, the swelling of neuronal cell bodies in control culture conditions is probably the mere consequence of iron-mediated ferroptosis. 

### 4.6. Nature of the Cell Death Process Prevented by TCs

The use of the fluorogenic probe DHR-123 revealed that in untreated cultures, ROS production becomes prominent in the subpopulation of neurons in which degenerative changes are in progress. Most interestingly, the increase in ROS was efficiently curtailed by **DOX**, **DDOX**, **DMC,** and **DDMC**, suggesting that ROS inhibition is pivotal for the rescue of DA neurons by neuroprotective TCs. Coherent with that, **CT**, which did not rescue DA neurons, also failed to inhibit oxidative stress under the present conditions. Overall, these data indicate that neuroprotective TCs act primarily by reducing oxidative stress-mediated insults that develop spontaneously and progressively in our cell culture paradigm.

APO and DES, which both possess iron-chelating properties, CAT, which detoxifies H_2_O_2,_ as well as TROL and LIP, which operates as a lipid peroxidation and ferroptosis inhibitor, respectively, were all capable of mimicking neuroprotective and ROS inhibitory effects of TCs. This indicates that neuroprotective TCs prevented the deleterious consequences of a sequence of events in which the reaction between Fe(II) and H_2_O_2_ (the Fenton reaction) generates hydroxyl radicals (^•^OH) [52], which in turn activates lipid peroxidation, a process characteristic of ferroptosis and PD neurodegeneration [53,54]. With APO and DES being specific chelators of Fe(III) [55,56], one may assume that redox cycling between Fe(II) and Fe(III) is a critical aspect of iron toxicity in this paradigm and that TCs may interfere in one way or another with this process. It should also be noted that the rescue effects provided by CAT, which is membrane impermeable [57], suggest that ^•^OH-mediated lipid peroxidation starts originally at the lipid–aqueous interface boundary of the plasma membrane. This might explain why the antioxidant VitC, which is effective in aqueous environments [58], could not mimic TC-mediated neuroprotection. 

In view of these results, we can assume that the protective effect of TCs was primarily to preserve the plasma membrane integrity, and secondarily to prevent the activation of downstream signaling events that cause intracellular propagation of oxidative stress through a mechanism of ROS-induced ROS release [59]. DHR-123, used for ROS monitoring in the present setting, is selectively trapped in mitochondria after its conversion by oxidation to a positively charged fluorescent dye, Rhodamine-123 [60]. One may therefore assume that protective TCs operate indirectly by preventing ROS emission by dysfunctional mitochondria.

### 4.7. Mechanisms Contributing to ROS Inhibition by TCs

With **DOX** and other TCs having the potential to form chelate complexes with iron [61,62], one may logically assume that the neuroprotective activity of this family of compounds was related to iron sequestration. For instance, **DOX** was reported to form a combination of 1:1 and 2:1 complexes with iron under physiological conditions [63]. With iron being present at a concentration of 1.6 μM in DF12 culture medium, **DOX** would be expected to provide optimal rescue to DA neurons between 1.6 and 3.2 μM, which was not the case, as neuroprotection with **DOX** under standard culture conditions was found to be optimal at 10 µM. Therefore, the chelating properties of **DOX,** and presumably of other TCs, are probably not solely responsible for the neuroprotective effects of these compounds in our model system. Accordingly, **DOX** was also reported to neutralize ROS in settings where iron mediation is not needed [64]. In particular, in cell free-systems, **DOX** has been found to scavenge superoxide anions and to prevent the formation of malondialdehyde (MDA) [64], a major by-product of the lipid peroxidation of cell membranes [65], having also the capacity to stimulate ROS production by mitochondria [66]. Therefore, TC-mediated neuroprotection might involve several mechanisms operating in a cooperative manner to prevent neuronal oxidative damage and ultimately neurodegeneration.

### 4.8. Neuroprotective TCs also Ensure Mitochondrial Health

By using DHR-123 together with TMRM, a cell-permeant dye that loads specifically in polarized (active) mitochondria [67,68], we were able to demonstrate that neuroprotective TCs as well as non-TC neuroprotectants prevent the decrease in ΔΨm occurring specifically in ROS-producing neurons. This observation signifies that TCs also preserved the integrity and function of mitochondria in these neurons, protecting them from energy failure and degeneration.

The present results are apparently coherent with data showing that TCs promote survival and fitness in mitochondrial disease models [33] by stimulating mitohormesis [33,69], i.e., a compensatory adaptive response to mild mitochondrial stress, wherein low, non-cytotoxic concentrations of ROS can serve as signaling molecules to initiate cellular events that ultimately protect cells from harmful effects [70]. However, such a mechanism is unlikely in the present paradigm, as neuronal ROS levels estimated in the presence of optimal concentrations of TCs remain low and similar to levels evaluated in APO-treated cultures.

The preservation of the mitochondrial function by TCs may rather depend, here, on the upstream inhibition of ROS. Interestingly, MDA, the major by-product of lipid peroxidation, was reported to promote both mitochondrial ROS production and ΔΨm dissipation [66], suggesting that TC may preserve mitochondrial fitness through their capacity to prevent oxidative degradation of lipids, primarily at the level of the plasma membrane. 

### 4.9. Neuroprotective TCs Are Ineffective against Acute Oxidative or Mitochondrial Insults

We also tested the efficacy of TCs against acute oxidative or mitochondrial insults induced by the oxidizing agent, H_2_O_2_, or the uncoupler of oxidative phosphorylation, FCCP, using midbrain cultures treated with APO to avoid the spontaneous demise of DA neurons.

Upon H_2_O_2_ exposure, we observed a robust increase in intracellular ROS correlated to a decrease in ΔΨm slightly greater than 30%, i.e., functional changes that closely mimic those observed in the course of spontaneous neurodegeneration. However, neither **DOX**, **DDOX**, **DMC,** nor **DDMC** reduced intracellular ROS production nor prevented mitochondrial membrane depolarization under these conditions, which certainly explains why TCs failed to afford protection in this paradigm. Note that this result can be probably explained by both the acuteness of this paradigm and the absence of scavenging properties of **DOX** for H_2_O_2_ [64].

Following FCCP exposure, we observed a large decrease in ΔΨm, as expected, and no significant elevation of ROS. This is somehow different from reports showing that uncoupling agents limit ROS production in isolated mitochondria [71,72], but in agreement with data showing that mitochondrial uncoupling has no effect on ROS production in isolated mitochondria oxidizing glucose-derived physiological substrates [73]. The inefficacy of **DOX**, **DDOX**, **DMC,** and **DDMC** to limit ΔΨm dissipation by FCCP explains without any doubt why TCs are not protective for DA neurons in this paradigm.

Overall, this set of data underlines the fact that TCs lose their neuroprotective efficacy when DA neurons experience acute oxidative and mitochondrial perturbations.

### 4.10. The Rescuing Effects of TCs Are Still Observable in Mature Midbrain Cultures

Finally, we wished to determine whether the neuroprotective effects of TCs for DA neurons remained observable when midbrain cultures endure low-level oxidative insults after an initial period of maturation in ACM to mimic as closely as possible brain physiopathological conditions in PD [19,74]. **DOX**, **DDOX**, **DMC,** and **DDMC** were robustly protective in this paradigm. Curiously, **DOX** appeared more potent in mature midbrain cultures than in younger ones, for a reason that we could not explain. This is, however, in line with a report showing that **DOX** efficiently protects DA neurons in a 6-OHDA rodent model of PD where oxidative stress-mediated neurodegeneration is progressive [10]. Most importantly, we found that all neuroprotective TCs preserved the uptake of DA under these culture conditions, indicating that the somato-dendritic and synaptic functions of DA neurons are also well maintained in TC-treated midbrain cultures [15,20].

**Figure 11:**
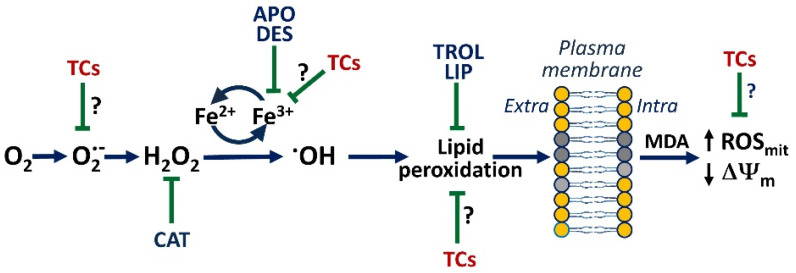
Schematic drawing showing how TCs may prevent the spontaneous loss of DA neurons induced by ferroptosis in midbrain culture. Under control conditions, soluble oxygen gives rise spontaneously to superoxide anions, which by dismutation produce H_2_O_2_ [75], which in turn is converted into ^•^OH through a Fenton-type reaction requiring Fe^2+^ as catalyst [52,75]. ^•^OH produced initially in the extracellular compartment stimulates lipid peroxidation on the outer side of the plasma membrane. The nature of downstream signaling events stimulating mitochondrial ROS production and promoting mitochondrial membrane depolarization remains to be established but MDA, a major by-product of lipid peroxidation of cell membranes [65], can produce both types of perturbations [66]. Neuroprotection by TCs could be due to superoxide scavenging, iron chelation, or the inhibition of lipid peroxidation propagation [63,64], with these mechanisms not mutually exclusive. The neuroprotective effects of TCs are mimicked by ferric iron chelators (APO; DES), which prevent iron **to** redox cycle, by the H_2_O_2_ detoxifying enzyme CAT, which inhibits the formation of ^•^OH, and by inhibitors of lipid peroxidation/ferroptosis (TROL, LIP), which protect the plasma membrane from the attack of ^•^OH. The effects of TCs are, however, not mimicked by VitC, a water-soluble antioxidant. The possibility that TCs could preserve mitochondrial health by stimulating mitohormesis [69] is not favored in the paradigm.

A schematic drawing that summarizes how TCs may protect DA neurons from iron-mediated oxidative stress insults is shown in Figure 11.

## 5. Conclusions

To conclude, we have established an in vitro system of mouse midbrain cultures for modeling chronic low-level oxidative stress mediated by iron as it may occur in PD neurodegeneration [8,16,17,18,53]. We showed that DA cell death is preventable by some TCs, independently of their antibiotic activity, through a mechanism that restrains oxidative stress-mediated insults and preserves the mitochondrial function. Although we are well aware that this model system cannot entirely recapitulate the complex and multifaceted nature of neurodegeneration in PD, we suggest that protective TCs, which lack antibiotic activity and reach the brain in relevant amounts may be of potential interest for PD treatment. This may be especially true for **DDMC**, which was also reported to inhibit αS aggregation [22], another important contributive mechanism to PD neurodegeneration.

## 6. Patents

The authors have filed patent applications for the use of non-antibiotic tetracycline derivatives for the treatment of Parkinson’s disease and related disorders.

## Figures and Tables

**Table 1:** Antimicrobial activity of TC derivatives.

TCs	*P. aeruginosa* PAO1	*E. coli* ATCC 25922	*S. aureus* ATCC 25923
	MIC (µM)		
**DDMC**	200	200	12.5
**DDOX**	>200	>200	>200
**DMC**	6.25	3.125	0.4
**CT**	6.25	6.25	1.6
**DOX**	12.5	3.125	0.4

## Data Availability

The datasets generated during the current study are available from the corresponding author upon reasonable request.
